# Geospatial methodology for determining the regional prevalence of hospital-reported childhood intussusception in patients from India

**DOI:** 10.1038/s41598-024-57187-8

**Published:** 2024-03-20

**Authors:** Shikha Dixit, Manoja Kumar Das, Durga Chitra Ramadugu, Narendra Kumar Arora, The INCLEN Intussusception Surveillance Network Study Group, Arindam Ray, Ashish Wakhlu, Bhadresh R. Vyas, Javeed Iqbal Bhat, Jayanta K. Goswami, John Mathai, K. Kameswari, Lalit Bharadia, Lalit Sankhe, M. K. Ajaya Kumar, Neelam Mohan, Pradeep K. Jena, Rachita Sarangi, Rashmi Shad, Sanjib K. Debbarma, J. Shyamala, Simmi K. Ratan, Suman Sarkar, Vijayendra Kumar, Anand P. Dubey, Atul Gupta, Bikasha Bihary Tripathy, Cenita J. Sam, Gowhar Nazir Mufti, Harsh Trivedi, Jimmy Shad, Kaushik Lahiri, Meera Luthra, P. Padmalatha, Rakesh Kumar, Ruchirendu Sarkar, A. Santosh Kumar, Subrat Kumar Sahoo, Sunil K. Ghosh, Sushant Mane, Bashir Ahmad Charoo, G. Rajendra Prasad, S. Harish Kumar, K. Jothilakshmi, Nihar Ranjan Sarkar, Pavai Arunachalam, Satya S. G. Mohapatra, Saurabh Garge

**Affiliations:** 1 The INCLEN Trust International, New Delhi, India; 2 Bill and Melinda Gates Foundation, India Country Office, New Delhi, India; 3 Department of Pediatric Surgery, King George’s Medical University, Uttar Pradesh, Lucknow, India; 4 Department of Pediatrics, MP Shah Government Medical College, Jamnagar, Gujarat India; 5 Department of Pediatrics, Sher-I-Kashmir Institute of Medical Sciences, Srinagar, Jammu & Kashmir India; 6 Department of Pediatric Surgery, Gauhati Medical College, Guwahati, Assam India; 7 Department of Pediatrics, PSG Institute of Medical Sciences, Coimbatore, Tamil Nadu India; 8 Department of Pediatric Surgery, Andhra Medical College, Vishakhapatnam, Andhra Pradesh India; 9 Fortis Escorts Hospital, Jaipur, Rajasthan India; 10 Department of Community Medicine, Grant Medical College and JJ Hospital, Mumbai, Maharashtra India; 11 Department of Pediatric Surgery, Government Medical College and SAT Hospital, Thiruvananthapuram, Kerala India; 12 The Medicity, Gurgaon, Haryana India; 13 Department of Pediatric Surgery, SCB Medical College, Cuttack, Odisha India; 14 Department of Pediatrics, IMS and SUM Medical College and Hospital, Bhubaneswar, Odisha India; 15 Choithram Hospital and Research Centre, Indore, Madhya Pradesh India; 16 Department of Pediatrics, Agartala Government Medical College, Agartala, Tripura India; 17 Apollo Hospitals, Chennai, Tamil Nadu India; 18 Department of Pediatric Surgery, Maulana Azad Medical College, Delhi, India; 19 Department of Pediatrics, Institute of Post Graduate Medical Education and Research, Kolkata, West Bengal India; 20 Department of Pediatric Surgery, Indira Gandhi Institute of Medical Sciences, Patna, Bihar India; 21 Department of Pediatrics, Maulana Azad Medical College, Delhi, India; 22 Department of Pediatric Surgery, IMS and SUM Medical College and Hospital, Bhubaneswar, Odisha India; 23 Department of Pediatric Surgery, PSG Institute of Medical Sciences, Coimbatore, Tamil Nadu India; 24 Department of Pediatric Surgery, Sher-I-Kashmir Institute of Medical Sciences, Srinagar, , Jammu & Kashmir India; 25 Department of Pediatric Surgery, MP Shah Government Medical College, Jamnagar, Gujarat India; 26 Department of Pediatrics, Andhra Medical College, Vishakhapatnam, Andhra Pradesh India; 27 Department of Pediatrics, Indira Gandhi Institute of Medical Sciences, Patna, Bihar India; 28 Department of Pediatric Surgery, Institute of Postgraduate Medical Education and Research, Kolkata, West Bengal India; 29 Department of Pediatrics, Government Medical College and SAT Hospital, Thiruvananthapuram, Kerala India; 30 Department of Pediatric Surgery, Agartala Government Medical College, Agartala, Tripura India; 31 Department of Pediatrics, Grant Medical College and JJ Hospital, Mumbai, Maharashtra India; 32 Department of Radiology, Institute of Post Graduate Medical Education and Research, Kolkata, West Bengal India; 33 Department of Radiology, IMS and SUM Medical College and Hospital, Bhubaneswar, Odisha India; 34 Masonic Medical Centre for Children, Coimbatore, India; 35 Department of Pediatrics, Neoclinic Children Hospital, Jaipur, India; 36 Department of Pediatric Surgery, All India Institute of Medical Sciences, Bhubaneswar, India

**Keywords:** Geospatial, GIS, Spatial clustering, Moran’s I, Hospital proximity, Intussusception, Children, India, Paediatrics, Diseases, Gastrointestinal diseases

## Abstract

Both developed and developing countries carry a large burden of pediatric intussusception. Sentinel site surveillance-based studies have highlighted the difference in the regional incidence of intussusception. The objectives of this manuscript were to geospatially map the locations of hospital-confirmed childhood intussusception cases reported from sentinel hospitals, identify clustering and dispersion, and reveal the potential causes of the underlying pattern. Geospatial analysis revealed positive clustering patterns, i.e., a Moran’s I of 0.071 at a statistically significant (*p* value < 0.0010) Z score of 16.14 for the intussusception cases across India (cases mapped n = 2221), with 14 hotspots in two states (Kerala = 6 and Tamil Nadu = 8) at the 95% CI. Granular analysis indicated that 67% of the reported cases resided < 50 km from the sentinel hospitals, and the average travel distance to the sentinel hospital from the patient residence was calculated as 47 km (CI 95% min 1 km–max 378 km). Easy access and facility referral preferences were identified as the main causes of the existing clustering pattern of the disease. We recommend designing community-based surveillance studies to improve the understanding of the prevalence and regional epidemiological burden of the disease.

## Introduction

Both developed and developing countries carry a large burden of pediatric intussusception^1^. The sentinel surveillance-based studies conducted thus far have highlighted the difference in the regional prevalence of intussusception in children, for example, in India, i.e., 17.7 (95% CI 5.9, 41.4) in northern India to 254 (95% CI 5.9, 41.4) cases per 100,000 child years in southern India^2,3^. However, the true incidence, prevalence and epidemiology of intussusception are unclear^3^. Community-based studies illustrating the distribution and referral pattern of patients with this acute abdominal emergency are limited^1,3^. INCLEN established a nationwide multisite sentinel surveillance network (23 hospitals) in India for documenting the epidemiology of intussusception in children during the pre- and postrotavirus vaccine introduction periods^4,5^.

Geospatial methods provide promising insights for analyzing the distribution, distance and density of health events, care-seeking behavior and referral patterns^6^. Disease mapping is one of the most important public health tools because it provides a comprehensive understanding of the relationships between health conditions and places^6–9^. Geographical information system (GIS)-based disease mapping helps in understanding the locations of disease occurrence, disease transmission patterns, environmental and socioeconomic risk factors, healthcare utilization and the spatial relationships between them^6,9^. Furthermore, geospatial tools utilizing residences and hospital locations/coordinates could provide useful insights into clients’ decision-making behavior and hospital catchment areas^6,9^. Healthcare research and service delivery rely heavily on GIS-based mapping and clustering technologies to improve the knowledge base and interpretation, as well as visualization of the population and risk factor characteristics^9–11^. Furthermore, GIS has been proven to be a very useful tool for healthcare facility planning, as it provides analytical tools for assessing facility catchments and objectively analyzing the distance between clients and hospitals using various matrices, e.g., straight lines, travel distances and travel times^12–15^. The geostatistical functions that were successfully utilized in epidemiology and public health studies include nearest neighbor analysis, spatial autocorrelation analysis, network analysis, location-allocation, quadrant analysis, distance matrix, hotspots, density, spatial interpolation and ordinary least squares regression^12–20^. However, the utilization of geospatial tools is limited in low- and middle-income countries (LMICs) due to the lack of standardized open source data at the granular scale, poor data currency and partial national spatial data infrastructure^21,22^. Thus, there are fewer research studies using geospatial approaches in LMICs than in high-income countries (HICs)^21–23^. The objectives of this article are to (1) illustrate the geospatial distribution of hospital-confirmed intussusception cases in children, (2) examine the statistically significant clustering and dispersion of the cases, and (3) assess the potential reasons behind the existing distribution patterns of the cases.

## Methods

The data utilized in this study were obtained from the INCLEN lead sentinel surveillance study^2,24^. The case data of intussusception children aged 2–23 months were collected from 23 nationally representative tertiary care hospitals (a mix of public and private) called sentinel surveillance sites across 22 states and union territories and were divided into four regions (North region, 5 sites, 3 public and 2 private; South region, 8 sites, 3 public and 5 private; East region, 7 sites, 6 public and 1 private; and West region, 3 sites, 2 public and 1 private) of India (S1 and S2; Supplementary file S1). The detailed methodology and selection of the sites have been published previously^5^. All of these sentinel hospitals served as referral centers for the same district, nearby districts, and even states. The INCLEN study documented intussusception in children aged 2–23 months through retrospective (from July 2010 to September 2017, total duration = 87 months) and prospective (April 2016–September 2017) surveillance conducted at these sentinel sites. The information on the address of the confirmed intussusception patients reported during the retrospective and prospective surveillance was collated for geospatial mapping at a granular scale.

### Tools and techniques

Geospatial analysis was conducted using GIS software, i.e., the Environmental System Research Institute (ESRI) and ArcGIS Desktop 10. 8.2 ©, 2021, and open source geospatial foundation project, Q-GIS 3.1©, 2020. The coordinates of the residential addresses were extracted using Google Earth Pro©. Coordinates (latitude and longitude) were taken in degree-minutes-second (DMS) format. All the coordinates were documented in the Google Sheet© for easy transfer to the GIS software for visualization and geoanalytics. Attribute data were prepared while maintaining the study participant’s unique IDs, and all other personal identifiers were removed. For the cases where the full address was not available, the subdistrict locations were used for the extraction of proxy address coordinates.

### Processes

**Figure 1:**
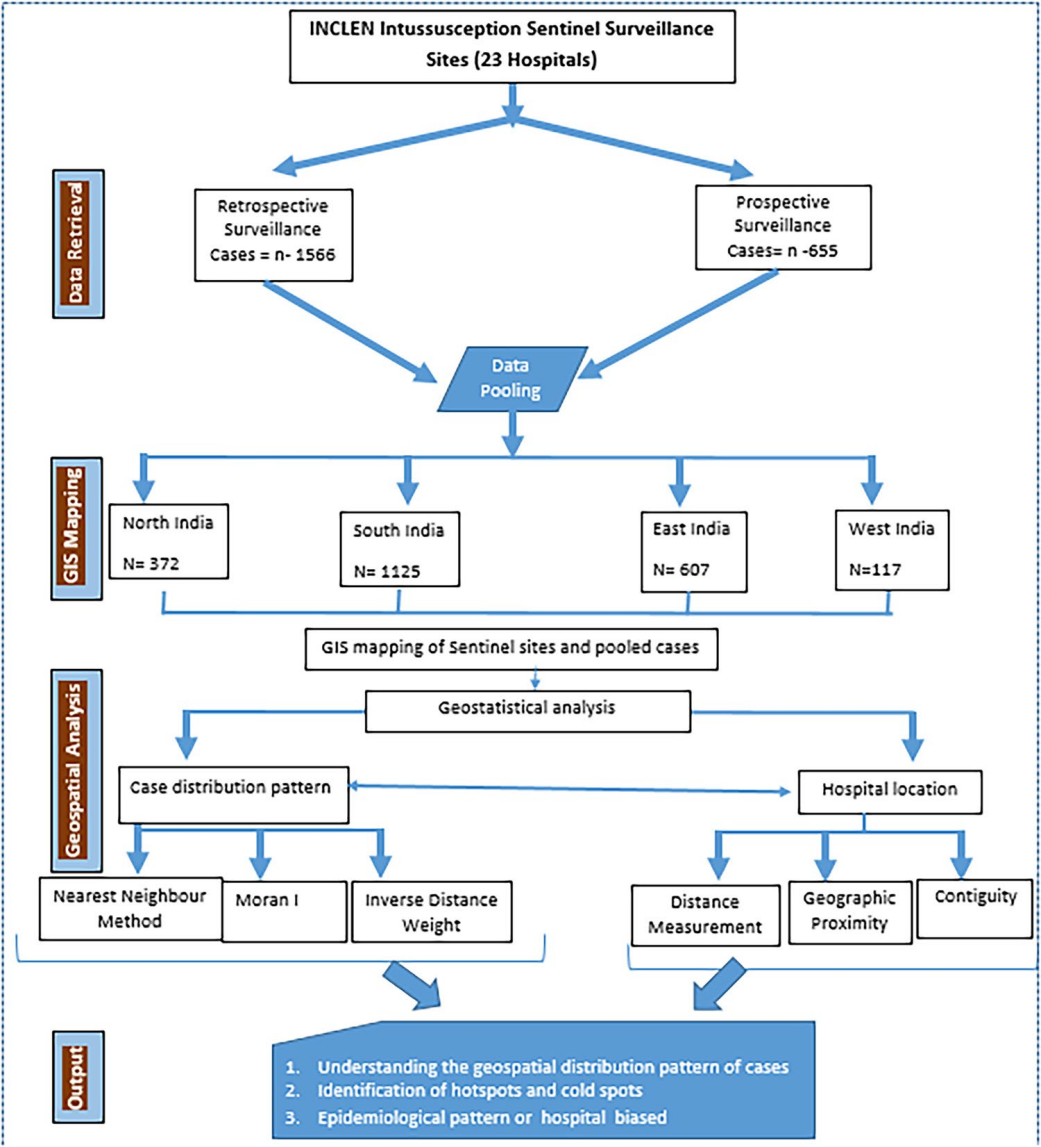
Study processes.

The retrospective and prospective surveillance data were pooled for geospatial mapping and analysis. The study process flow is shown in Fig. 1. The findings generated through the descriptive analysis are expressed as proportions, means, standard deviations, or medians and interquartile ranges (IQRs), as appropriate. The specific geostatistical data analyses were conducted for the point-pattern analysis, geographical proximity, and care-seeking phenomena, as mentioned below.

- *The nearest neighbor index* (NNI) was used to precisely measure the spatial relationship between the distribution of intussusception cases according to their residence^13,25^.
- *Moran’s I* was calculated using the Global Moran's I tool to document the spatial autocorrelation based on the location and density of the cases. The z score and p value for statistical significance were calculated.
- *Hotspot analysis* was performed using the Getis-Ord Gi* statistic after confirmation of the clustering in the data for each point feature (case location). For hotspot analysis, the data were processed using an integrated tool, and each point was given weights (inverse distance weights) based on the number of cases colliding within a 5-km diameter. The diameter was fixed by moving the circular window via the iterative distance method. The hotspot analysis helped in the identification of hot spots and cold spots using statistical significance.
- *Inverse distance weighting (IDW):* The z scores were derived from the hotspot technique for visualization of the multivariate interpolation using the IDW function. The IDW assigned values to unknown points and prepared a continuous surface using a weighted average.
- *Geographical proximity of the treating facility and contiguity:* The distance from the patient’s residence to the treating hospital was calculated using the hub analysis function in QGIS 3.1. Overlaying analysis was conducted using ArcGIS 10.8.2 software. Distribution pattern of patient residences according to hospital location studied using GIS tools, i.e., buffer, locational, contiguity analysis, etc.
- *A hub analysis and distance matrix* were used to calculate the distance (Euclidean distance) of the treating hospitals from the residence. The cases were categorized into five distance bands: < 10 km, > 10–50 km, > 50–100 km, > 100–250 km, and > 251 km^13,25^.

### Ethical issues

The study protocol was reviewed and approved by the ethics review committees of all the participating institutes. Confidentiality in the data handling was maintained. All the methods were performed in accordance with the ICMR National Ethical Guidelines for Biomedical and Health Research (2017).

### Ethics approval and consent to participate

The study protocol was reviewed and approved by all the participating institutes. The list of ethics committees of the participating institutes included the INCLEN Independent Ethics Committee, The INCLEN Trust International, New Delhi, India (Ref No: IIEC 23; Dated June 30, 2015); the Institutional Ethics Committee, King George’s Medical University, Lucknow, Uttar Pradesh, India (Ref no: 7951/Ethics/R. Cell-15; Dated December 4, 2015); Institutional Ethics Committee, MP Shah Government Medical College, Jamnagar, Gujarat, India (ref no: 01/46/2016; Dated January 6, 2016); Institutional Ethics Committee, Sher-I-Kashmir Institute of Medical Sciences, Srinagar, Jammu & Kashmir, India (Ref no: 42/2015; Dated October 29, 2015); Institutional Ethics Committee, Gauhati Medical College, Guwahati, Assam, India (Ref no: MC/02/2015/274; Dated May 30, 2016); Institutional Human Ethics Committee, PSG Institute of Medical Sciences, Coimbatore, Tamil Nadu, India (Ref no 15/294; Dated November 16, 2015); Institutional Ethics Committee, King George Hospital, Andhra Medical College, Vishakhapatnam, Andhra Pradesh, India (Dated October 26, 2015); Institutional Ethics Committee, Fortis Escorts Hospital, Jaipur, Rajasthan, India (Ref no: FEHJ/IEC/15/0023; Dated September 14, 2015); Institutional Ethics Committee, Grant Medical College & JJ Hospital, Mumbai, Maharashtra, India (Ref no: IEC/Pharm/288/15; Dated November 19, 2015); Institutional Ethics Committee, Government Medical College & SAT Hospital, Thiruvananthapuram, Kerala, India (Ref no: 06/05/2015/MCT; Dated December 9, 2015); Medanta Institutional Ethics Committee, Medanta-The Medicity, Gurgaon, Haryana, India (MICR 559/2015; Dated January 21, 2016); Institutional Ethics Committee, SCB Medical College, Cuttack, Odisha, India; Institutional Ethics Committee, IMS & SUM Medical College & Hospital, Bhubaneswar, Odisha, India (Ref no 210/5/10/2015; Dated October 14, 2015); Institutional Ethics Committee; Choithram Hospital and Research Centre, Indore, Madhya Pradesh, India (Ref no: EC/Oct/15/20; Dated October 27, 2015); Institutional Ethics Committee, Agartala Government Medical College, Agartala, Tripura, India; Institutional Ethics Committee- Clinical Studies, Apollo Hospitals, Chennai, Tamil Nadu (Dated October 14, 2015); Institutional Ethics Committee, Maulana Azad Medical College, Delhi, India (Ref no: F.1/IEC/MAMC/50/4/2015/308; Dated November 20, 2015); Institutional Ethics Committee, Institute of Post Graduate Medical Education and Research, Kolkata, West Bengal, India (Ref no: Inst/IEC/2016/197; Dated March 1, 2016); Institutional Ethics Committee, Indira Gandhi Institute of Medical Sciences, Patna, Bihar, India (Ref no: 1256/Acad; Dated November 11, 2016); and Ethics Committee, Apollo Hospital, Hyderabad, Telengana, India ((Dated October 13, 2015). The interviews with stakeholders were performed after written informed consent was obtained. Written informed consent was obtained from the parents/legal guardians of the children enrolled in the study.

## Results

### Study participants and demographics

During the surveillance period, 2279 episodes of intussusception were reported among 2221 children across the 23 network hospitals (S1, S2; Supplementary file S1). A total of 58 children out of 2221 had more than 1 episode of hospitalization for intussusception. Thus, data for 2221 children (retrospective surveillance, n = 1566; prospective surveillance, n = 655) were analyzed. There were 1467 (66%) boys and 754 (34%) girls (male‒female ratio, 1.96). The majority (75%) of patients were less than 12 months old. A sharp increase (fivefold increase) in the annual number of intussusception cases at the sites was observed from 2010 to 2017 (S3, Supplementary file S1), with the highest (31%) increase occurring between 2014 and 2015.

### Pattern and clustering of cases

**Figure 2:**
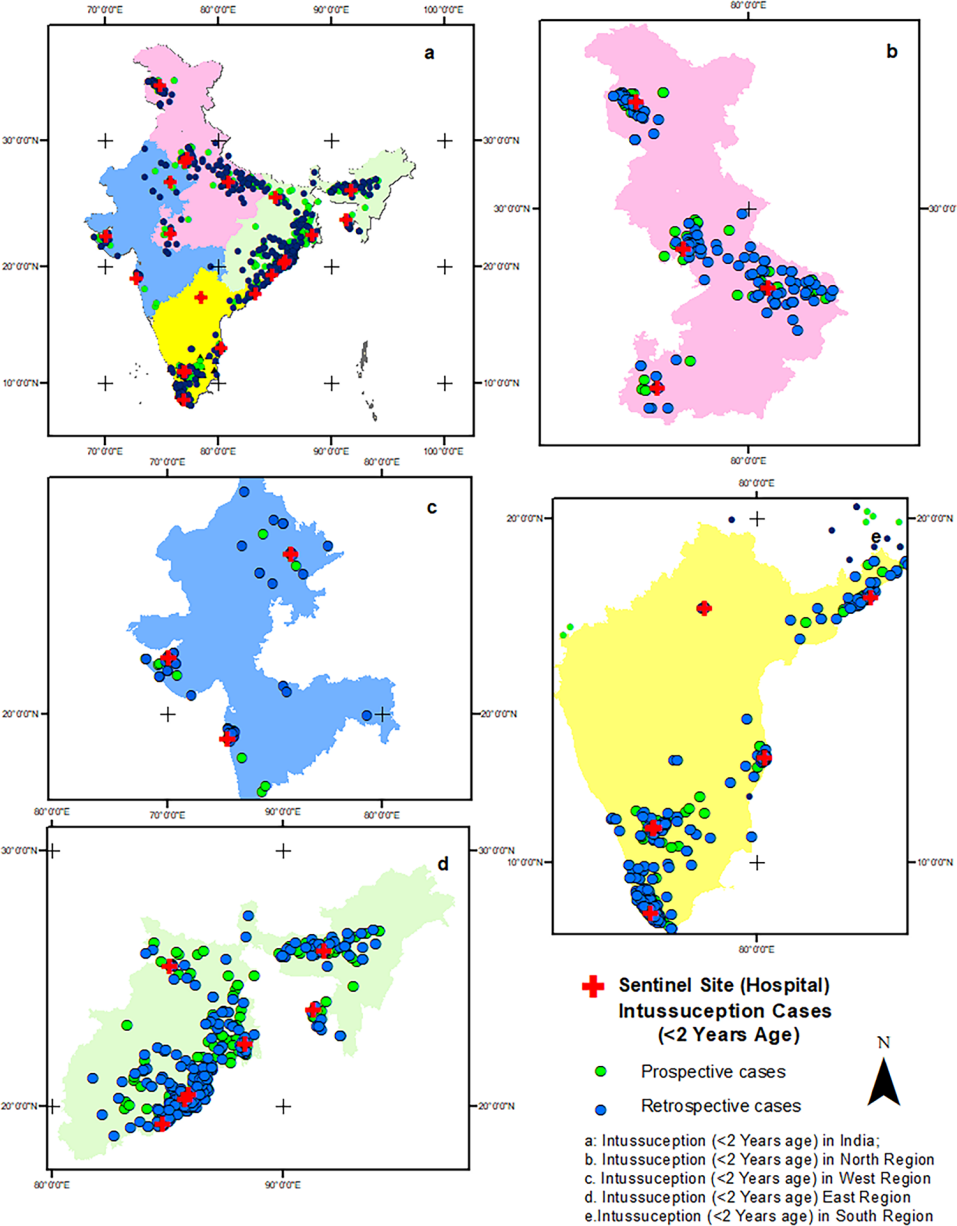
Distribution of intussusception cases. (**a**) Country-level distribution of patients. (**b**) Spatial pattern of intussusception cases and sentinel site hospital location in the northern region. (**c**) Spatial pattern of intussusception cases and sentinel site hospital location in the western region. (**d**) Spatial pattern of intussusception cases and sentinel site hospital location in the eastern region. (**e**) Spatial pattern of intussusception cases and sentinel site hospital location in the southern region. Software used: Arc GIS 10.8.2 https://desktop.arcgis.com/en/arcmap.

Throughout the surveillance period, the highest number of intussusception cases was reported from the southern region (n = 1125, 51%; retrospective n = 833, prospective, n = 292), followed by the eastern (n = 607, 26.4%; retrospective, n = 410, prospective, n = 197), northern (n = 372, 17.1%; retrospective, n = 239, prospective, n = 133) and western (n = 117, 5.3%; retrospective, n = 84, prospective, n = 33) regions. The region wise case distribution pattern is shown in Fig. 2.

The average NNI was 0.171 (*p* value < 0.001) according to the pooled cases, suggesting a highly clustered pattern. The NNI varied from 0.311 to 0.171 across the four regions. The clustering pattern was further confirmed by a Moran’s I index of 0.071 at a statistically significant (*p* value < 0.0010) z score of 16.14. Moran’s I indicates the location of statistically significant hotspots in a few locations.

### Hotspot analysis and IDW

**Figure 3:**
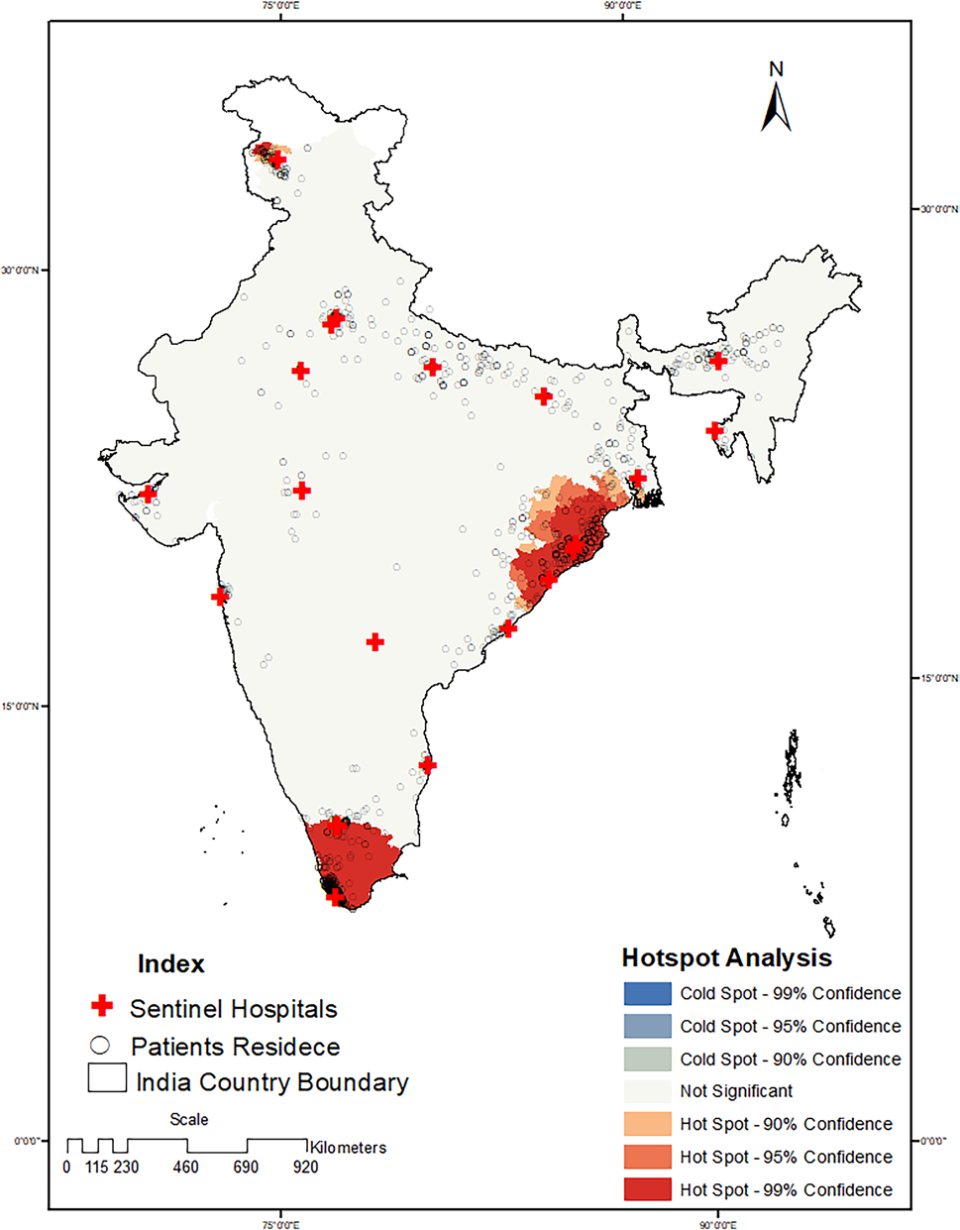
Hotspot analysis of the residence location of children under two years old with intussusception in India. Software used: Arc GIS 10.8.2 https://desktop.arcgis.com/en/arcmap.

**Figure 4:**
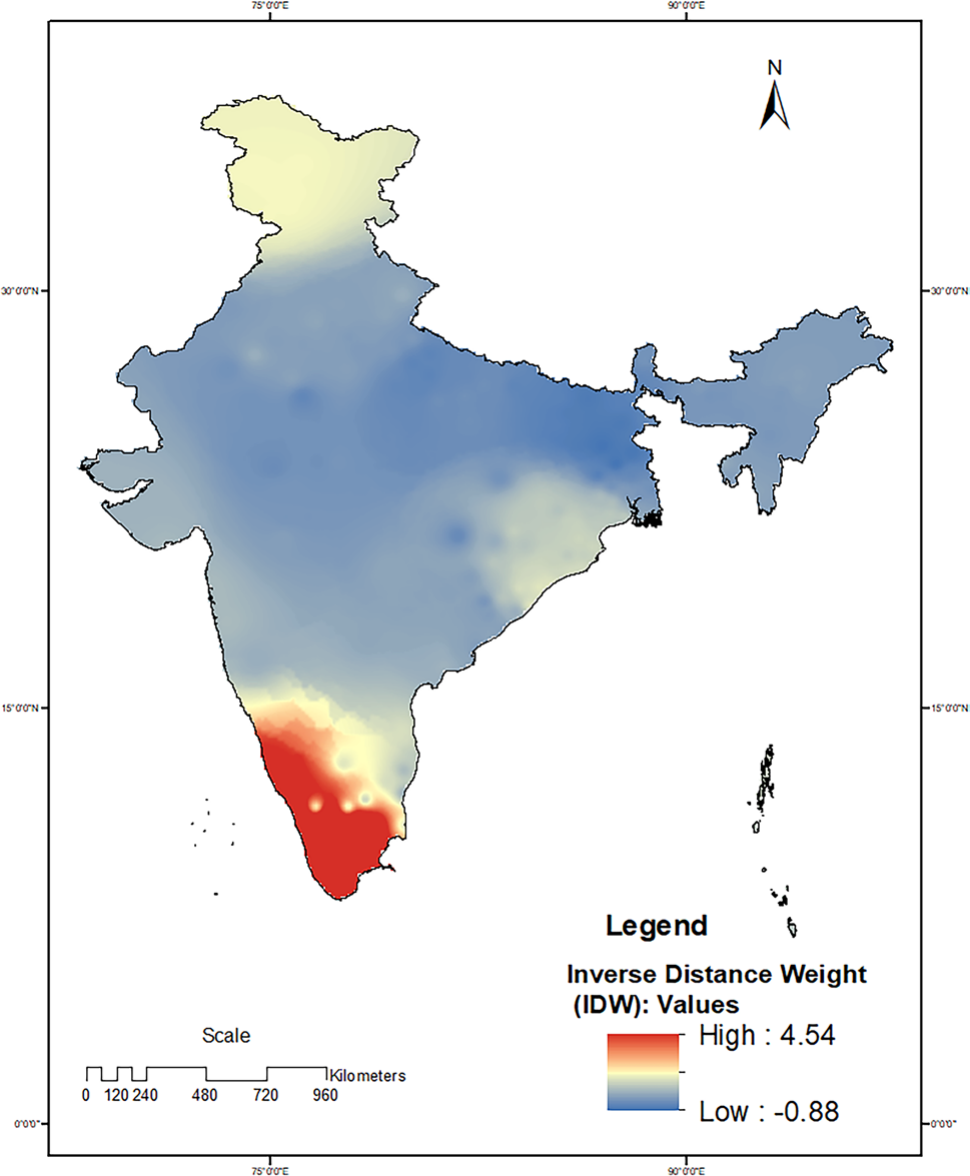
Interpolation of hotspots (z scores) and inverse distance weights (IDWs) for the residence locations of children under two years old with intussusception in India. Software used: Arc GIS 10.8.2 https://desktop.arcgis.com/en/arcmap.

The three regions where disease hotspots were found were the southern region, followed by the eastern and the northern regions. Hotspots were located in the Trivandrum (Kerala), Cuttack (Odisha), and Srinagar (Jammu and Kashmir) districts of India (see Fig. 3). Granular analysis revealed that, out of the 2221 locations (residence of children with intussusception) across the country, a high density of cases was reported from 14 locations (6 in Kerala and 8 in Tamil Nadu). The IDW surface generated using z scores computed via hotspot analysis indicated that the surface area followed a locus pattern and that the IDW faded with increasing distance from the hospital (Fig. 4). Additionally, the IDW values varied from 4.45 to –0.88 across the sites, which indicated greater variation and clustering.

### Hub analysis and distance matrix

The average distance traveled by families to access a hospital for intussusception treatment was 47 km (95% CI 1–378). At the pooled level, 67% of the patients resided less than 50 km from the hospitals. No preferential behavior is observed in terms of traveling long distances for the treatment of male versus female children. Table 1 shows the distribution of the patients according to the different distance bands from the hospitals across all the 4 regions.

**Table 1:** Region wise distribution of the patient’s residence (in %) falling in different distance bands from the treating hospitals.

Region	< 10 km	10–50 km	50–100 km	101–250 km	> 251 km	Episodes (n) reported during the surveillance timeline
Distance of patient’s residence from the treating hospital (% of total cases in different distance bands)						
East	15.8	31.6	26.3	24.7	1.9	582
North	18.3	35.7	24.2	20.8	1.0	389
South	31.0	50.8	12.3	5.4	0.4	1167
West	45.4	21.3	12.8	17.7	2.8	141
Pooled (n)	589	946	409	313	22	2279

The numbers indicate the number of hospitalized intussusception episodes in children (n = 2279).

### Distribution of cases and hospital proximity

The majority (n = 1555, 70%) of the reported cases were concentrated around six network hospitals. Thiruvananthapuram (Kerala, n = 843, 36.9%), Cuttack (Odisha, n = 271, 11.8%) and Srinagar (Jammu & Kashmir, n = 153, 6.7%) contributed the highest number of cases. The proximity analysis conducted in the GIS environment revealed that the majority of the cases were from nearby areas (same or adjacent districts). This was especially the case in the southern and western regions, where 59.4% and 67.8%, respectively, of the patients were concentrated at the same hospital or adjacent districts (Table 2).

**Table 2:** The distribution of intussusception cases in children under two years of age as per the location of treating hospital.

Region	Location of the patients vis-à-vis treating hospital location		
	Same district, n (%)	Adjacent district, n (%)	Other (far) districts, n (%)
East	158 (26.3)	211 (35.1)	232 (38.6)
North	108 (27.7)	105 (26.9)	177 (45.4)
South	693 (59.4)	395 (33.8)	79 (6.8)
West	82 (67.8)	14 (11.6)	25 (20.7)
Pooled*	1041(45.68)	725(31.81)	513 (22.51)

*The numbers indicate the hospitalized intussusception episodes in the children (n-2279).

## Discussion

This is the first study describing the detailed spatial distribution of intussusception cases among children aged less than 2 years in India. The spatial aspects of the intussusception cases, including their geographical distribution, distance, density and direction, are presented in this manuscript. Previous studies have reported that the epidemiology of intussusception varies according to the country and region^2,26^. However, granular examination of the location of patients reported at sentinel surveillance network hospitals, with the help of geostatistics techniques, revealed that the regional pattern inferred from hospital-based studies may be misleading. Due to reporting bias, unfixed catchments, and unavailability of clinical registries, hospital caseloads do not represent the true epidemiological burden of the disease. It is important to note that the healthcare services offered by 23 hospitals under the surveillance site network were highly specialized, and admission to these hospitals was strictly referral-based^5,24^. Furthermore, a large proportion of patients with pediatric intussusception conditions do not reach tertiary care hospitals, resort to local healthcare facilities, reach tertiary healthcare facilities late, or die without timely intervention^1^. Other studies have also reported differential case fatalities, as observed in Africa (1 death in every 10 hospital admissions) and the rest of the world (fewer than 1 death in every 100–2000 hospital admissions)^3,27^. The availability of pediatric surgical facilities, specialists in surveillance network hospitals, referral systems and accessibility were the major factors influencing the differential caseloads reported from the surveillance hospitals. Understanding and recognizing the causes of disease localization are important in assessing the regional differences in the number of intussusception cases.

Like many other LMICs, India lacks a centralized health administrative database and comprehensive clinical registries^28^. Although the sentinel hospitals were tertiary care hospitals serving large catchment areas, there were other hospitals also serving the same catchment area. In the absence of fixed catchment areas, estimation of the risk ratio, floating accessibility^14,15^, demand and supply could not be performed. The catchment area of each hospital is influenced by the distance. The six hospitals from the three regions, i.e., southern (n-2), eastern (n-2) and northern (n-2) regions, had the highest number of cases. A total of 86% (1904/2221) of the episodes were reported from subdistricts within 100 km of referral hospitals. Approximately 60% of the patients in the southern region were from the same district, and 34% of the patients were from other districts where the hospitals were based. Conversely, in the North region, 45% of the patients were from distant districts (neither from the same district nor from adjacent districts). India is a large country with a 3.2 million km^2^ area. The probable reason for the greater average distance traveled in the North region is the relatively poor density of hospitals compared to that in the South region. However, further exploration is needed to determine the reasons for this interesting pattern of hospital access in the southern region, where disease reporting at sentinel hospitals was mostly from nearby areas, versus the northern region of India, where patients were from faraway areas.

The probability of cases being reported at hospitals for surgical purposes is a function of the distance between the patient location and healthcare providers^29^. The case reporting probability decreases (0.1–0.6) at a travel distance of 10 km and is effectively zero at distances > 30–40 km^29^. Our analysis clearly showed that the number of cases and hospital distance were inversely related, as^29^ two-thirds (67%) of the cases reported at the network hospitals were located less than 50 km apart, while one-third (33%) traveled more than 50 km. The median distance traveled for an intussusception patient was 47 km (95% CI 1–378 km) for hospitalization.

As per the 2011 census conducted by the national government, there are 640 districts and 5570 subdistricts in India. In this study, three major hotspots were identified, covering only 12 (n-640) districts of India. Techniques utilized for measuring the spatial pattern of diseases, i.e., nearest neighbor analysis (NNA), Moran's I, and disease hotspots, were also used in the literature^10,18–20^. Spatial analysis proved to be a valuable method for exploring the spatial patterns of intussusception cases.

Although demographic, clinical, diagnostic and treatment practice-related data were available in the hospital logs and registers, information on the patient distance and mode of transport road conditions was missing. Another constraint in conducting sophisticated network analysis is the unavailability of base maps depicting the road network with road type, surface, and quality information. Additionally, in LMICs, there is no well-established or functioning public transport system in many areas of developing countries^13^, and roads are adopted because of the convenience of traveling on foot or by vehicle. These data-related constraints are not found in the case of HICs^14,15^. The calculation of straight-line distances in a GIS environment provided a useful alternative to fill this data gap, and objective measures were made on the distance variable. The distance matrix used by us required only two-point coordinates, i.e., the origin (patient home) to the destination (health care facility) and a georeferenced baseman. A strong correlation was noted between straight-line distances and travel distances (r = 0.92, (p < 0.001))^13^. Euclidean distance was considered an acceptable proxy for the time spent traveling, especially in resource-poor settings^13^. For some locations, complete addresses were missing in the records; therefore, density clusters were generalized up to the subdistrict scale (1:5000 m). Patients who fell within a 5 km distance were snapped during the preprocessing stage. The 5 km radius was selected through the iterative process considering subdistricts as a primary unit of analysis.

Active surveillance or community-based surveillance provides a more complete picture for estimating the true burden of diseases^29^. Nevertheless, the strength of this study is that it provides important insights into the geospatial distribution of intussusception cases and the potential causes of the emerging pattern. To suggest any epidemiological pattern, more expanded regional and community-based studies are required to validate the results. Community-based studies conducted at demographic and health surveillance sites can provide added advantages by providing the exact population denominator for the calculation of spatial and temporal disease rates.

## Limitations

This study has several limitations. Approximately 70% (2279) of the cases were captured through retrospective surveillance, and background information about these patients was retrieved from the hospital records. The data about barriers to seeking healthcare, such as socioeconomic status, access (travel mode), route, and time, were not available in hospital records. Furthermore, sentinel surveillance failed to provide a community-level estimation of total intussusception cases because there were more hospitals in the districts where patients might have gone for treatment, but cases could not be considered. The distance between the residence and the hospital was calculated using the straight-line method in a GIS environment. However, the exact travel-time distance could not be calculated using network analysis due to the lack of data on the travel mode and route taken by the patients. Granular data on road networks, including level, surface typology, and quality, were not available in open-source libraries. Additionally, the analysis was limited by the inability to define the catchment area for intussusception cases and to obtain accurate birth cohort data for the catchment population.

## Conclusions

This study contributes new knowledge on the epidemiology of intussusception in children under two years of age and can reveal the regional pattern of intussusception, which cannot be determined by merely performing a quantitative analysis of cases from these sentinel sites. The use of sentinel surveillance data alone has health facility bias because the concentration of cases will increase near the preferred facility. Therefore, to assess the true burden and regional pattern of this disease, community-based surveillance studies are required at the identified hotspots and cold spot areas. Further research, including detailed information on local healthcare-seeking behavior, along with population measurements, is needed for accurate estimation of population-based incidence rates and epidemiology.

### Supplementary Information

Supplementary Information.

## Data Availability

All the data are available from the investigators and can be provided by the corresponding author upon reasonable request.

## Abbreviations

GIS: Geographical information system
km: Kilometer
NNI: Nearest Neighbor Index
LMIC: Low and middle income country
HIC: High income country
