# Supplementary material for: Geospatial methodology for determining the regional prevalence of hospital-reported childhood intussusception in patients from India
Source: Sci Rep. 2024 Mar 20;14:6664. doi: 10.1038/s41598-024-57187-8 (PMC10954623; doi:10.1038/s41598-024-57187-8)
Supplement: Supplementary file 1 — Supplementary Information. [file 41598_2024_57187_MOESM1_ESM.docx]

**Supplementary Table S1: State wise distribution of Intussusception cases**

| **Sl No** | **State Name** | **Retrospective Cases** | **Prospective Cases** | **Total** |
| --- | --- | --- | --- | --- |
| 1 | Andhra Pradesh | 56 | 24 | 80 |
| 2 | Assam | 82 | 34 | 116 |
| 3 | Bihar | 11 | 9 | 20 |
| 4 | Chhattisgarh | 1 | 0 | 1 |
| 5 | Delhi | 45 | 11 | 56 |
| 6 | Gujarat | 36 | 21 | 57 |
| 7 | Haryana | 10 | 6 | 16 |
| 8 | Jammu & Kashmir | 80 | 73 | 153 |
| 9 | Jharkhand | 1 | 1 | 2 |
| 10 | Karnataka | 2 | 0 | 2 |
| 11 | Kerala | 646 | 187 | 833 |
| 12 | Madhya Pradesh | 13 | 6 | 19 |
| 13 | Maharashtra | 28 | 7 | 35 |
| 14 | Meghalaya | 1 | 0 | 1 |
| 15 | Nepal* | 1 | 0 | 1 |
| 16 | Odisha | 269 | 104 | 373 |
| 17 | Rajasthan | 23 | 5 | 28 |
| 18 | Sikkim | 1 | 0 | 1 |
| 19 | Tamilnadu | 154 | 89 | 243 |
| 20 | Tripura | 8 | 9 | 17 |
| 21 | Uttar Pradesh | 101 | 39 | 140 |
| 22 | Uttarakhand | 1 | 0 | 1 |
| 23 | West Bengal | 39 | 45 | 84 |
|  | **Total** | **1609** | **670** | **2279** |

*One case from Nepal reported at the network hospital

| **S.No** | **Months** | **Years**  **Supplementary Table S2: Site wise temporal pattern of intussusception cases** | **Month/ year** | **Zone** | **Site codes** | **Site Name** | **No of Cases** | **Male** | **Female** |
| --- | --- | --- | --- | --- | --- | --- | --- | --- | --- |
|  | **7** | **2010** | **Jul/2010** | **South** | **9** | Vishakhapatanam | 1 | 0 | 1 |
|  | **7** | **2010** | **Jul/2010** | **South** | **10** | Hyderabad | 0 | 0 | 0 |
|  | **7** | **2010** | **Jul/2010** | **South** | **11** | Coimbatore | 1 | 1 | 0 |
|  | **7** | **2010** | **Jul/2010** | **South** | **12** | Chennai | 1 | 1 | 0 |
|  | **7** | **2010** | **Jul/2010** | **South** | **13** | Trivandrum | 7 | 3 | 4 |
|  | **8** | **2010** | **Aug/2010** | **South** | **9** | Vishakhapatanam | 0 | 0 | 0 |
|  | **8** | **2010** | **Aug/2010** | **South** | **10** | Hyderabad | 0 | 0 | 0 |
|  | **8** | **2010** | **Aug/2010** | **South** | **11** | Coimbatore | 0 | 0 | 0 |
|  | **8** | **2010** | **Aug/2010** | **South** | **12** | Chennai | 1 | 0 | 1 |
|  | **8** | **2010** | **Aug/2010** | **South** | **13** | Trivandrum | 13 | 12 | 1 |
|  | **9** | **2010** | **Sep/2010** | **South** | **9** | Vishakhapatanam | 0 | 0 | 0 |
|  | **9** | **2010** | **Sep/2010** | **South** | **10** | Hyderabad | 0 | 0 | 0 |
|  | **9** | **2010** | **Sep/2010** | **South** | **11** | Coimbatore | 0 | 0 | 0 |
|  | **9** | **2010** | **Sep/2010** | **South** | **12** | Chennai | 0 | 0 | 0 |
|  | **9** | **2010** | **Sep/2010** | **South** | **13** | Trivandrum | 4 | 1 | 3 |
|  | **10** | **2010** | **Oct/2010** | **South** | **9** | Vishakhapatanam | 1 | 0 | 1 |
|  | **10** | **2010** | **Oct/2010** | **South** | **10** | Hyderabad | 0 | 0 | 0 |
|  | **10** | **2010** | **Oct/2010** | **South** | **11** | Coimbatore | 2 | 0 | 2 |
|  | **10** | **2010** | **Oct/2010** | **South** | **12** | Chennai | 0 | 0 | 0 |
|  | **10** | **2010** | **Oct/2010** | **South** | **13** | Trivandrum | 8 | 6 | 2 |
|  | **11** | **2010** | **Nov/2010** | **South** | **9** | Vishakhapatanam | 0 | 0 | 0 |
|  | **11** | **2010** | **Nov/2010** | **South** | **10** | Hyderabad | 0 | 0 | 0 |
|  | **11** | **2010** | **Nov/2010** | **South** | **11** | Coimbatore | 1 | 1 | 0 |
|  | **11** | **2010** | **Nov/2010** | **South** | **12** | Chennai | 2 | 2 | 0 |
|  | **11** | **2010** | **Nov/2010** | **South** | **13** | Trivandrum | 7 | 4 | 3 |
|  | **12** | **2010** | **Dec/2010** | **South** | **9** | Vishakhapatanam | 0 | 0 | 0 |
|  | **12** | **2010** | **Dec/2010** | **South** | **10** | Hyderabad | 0 | 0 | 0 |
|  | **12** | **2010** | **Dec/2010** | **South** | **11** | Coimbatore | 0 | 0 | 0 |
|  | **12** | **2010** | **Dec/2010** | **South** | **12** | Chennai | 0 | 0 | 0 |
|  | **12** | **2010** | **Dec/2010** | **South** | **13** | Trivandrum | 8 | 4 | 4 |
|  | **1** | **2011** | **Jan/2011** | **South** | **9** | Vishakhapatanam | 0 | 0 | 0 |
|  | **1** | **2011** | **Jan/2011** | **South** | **10** | Hyderabad | 0 | 0 | 0 |
|  | **1** | **2011** | **Jan/2011** | **South** | **11** | Coimbatore | 1 | 1 | 0 |
|  | **1** | **2011** | **Jan/2011** | **South** | **12** | Chennai | 3 | 2 | 1 |
|  | **1** | **2011** | **Jan/2011** | **South** | **13** | Trivandrum | 8 | 4 | 4 |
|  | **2** | **2011** | **Feb/2011** | **South** | **9** | Vishakhapatanam | 0 | 0 | 0 |
|  | **2** | **2011** | **Feb/2011** | **South** | **10** | Hyderabad | 0 | 0 | 0 |
|  | **2** | **2011** | **Feb/2011** | **South** | **11** | Coimbatore | 2 | 2 | 0 |
|  | **2** | **2011** | **Feb/2011** | **South** | **12** | Chennai | 6 | 5 | 1 |
|  | **2** | **2011** | **Feb/2011** | **South** | **13** | Trivandrum | 7 | 6 | 1 |
|  | **3** | **2011** | **Mar/2011** | **South** | **9** | Vishakhapatanam | 0 | 0 | 0 |
|  | **3** | **2011** | **Mar/2011** | **South** | **10** | Hyderabad | 0 | 0 | 0 |
|  | **3** | **2011** | **Mar/2011** | **South** | **11** | Coimbatore | 1 | 1 | 0 |
|  | **3** | **2011** | **Mar/2011** | **South** | **12** | Chennai | 1 | 0 | 1 |
|  | **3** | **2011** | **Mar/2011** | **South** | **13** | Trivandrum | 16 | 10 | 6 |
|  | **4** | **2011** | **Apr/2011** | **South** | **9** | Vishakhapatanam | 2 | 2 | 0 |
|  | **4** | **2011** | **Apr/2011** | **South** | **10** | Hyderabad | 0 | 0 | 0 |
|  | **4** | **2011** | **Apr/2011** | **South** | **11** | Coimbatore | 1 | 0 | 1 |
|  | **4** | **2011** | **Apr/2011** | **South** | **12** | Chennai | 2 | 1 | 1 |
|  | **4** | **2011** | **Apr/2011** | **South** | **13** | Trivandrum | 9 | 5 | 4 |
|  | **5** | **2011** | **########** | **South** | **9** | Vishakhapatanam | 1 | 0 | 1 |
|  | **5** | **2011** | **########** | **South** | **10** | Hyderabad | 0 | 0 | 0 |
|  | **5** | **2011** | **########** | **South** | **11** | Coimbatore | 0 | 0 | 0 |
|  | **5** | **2011** | **########** | **South** | **12** | Chennai | 1 | 1 | 0 |
|  | **5** | **2011** | **########** | **South** | **13** | Trivandrum | 9 | 5 | 4 |
|  | **6** | **2011** | **Jun/2011** | **South** | **9** | Vishakhapatanam | 0 | 0 | 0 |
|  | **6** | **2011** | **Jun/2011** | **South** | **10** | Hyderabad | 0 | 0 | 0 |
|  | **6** | **2011** | **Jun/2011** | **South** | **11** | Coimbatore | 1 | 1 | 0 |
|  | **6** | **2011** | **Jun/2011** | **South** | **12** | Chennai | 1 | 0 | 1 |
|  | **6** | **2011** | **Jun/2011** | **South** | **13** | Trivandrum | 9 | 3 | 6 |
|  | **7** | **2011** | **Jul/2011** | **South** | **9** | Vishakhapatanam | 0 | 0 | 0 |
|  | **7** | **2011** | **Jul/2011** | **South** | **10** | Hyderabad | 0 | 0 | 0 |
|  | **7** | **2011** | **Jul/2011** | **South** | **11** | Coimbatore | 0 | 0 | 0 |
|  | **7** | **2011** | **Jul/2011** | **South** | **12** | Chennai | 0 | 0 | 0 |
|  | **7** | **2011** | **Jul/2011** | **South** | **13** | Trivandrum | 3 | 2 | 1 |
|  | **8** | **2011** | **Aug/2011** | **South** | **9** | Vishakhapatanam | 1 | 1 | 0 |
|  | **8** | **2011** | **Aug/2011** | **South** | **10** | Hyderabad | 0 | 0 | 0 |
|  | **8** | **2011** | **Aug/2011** | **South** | **11** | Coimbatore | 2 | 0 | 2 |
|  | **8** | **2011** | **Aug/2011** | **South** | **12** | Chennai | 3 | 2 | 1 |
|  | **8** | **2011** | **Aug/2011** | **South** | **13** | Trivandrum | 6 | 5 | 1 |
|  | **9** | **2011** | **Sep/2011** | **South** | **9** | Vishakhapatanam | 1 | 0 | 1 |
|  | **9** | **2011** | **Sep/2011** | **South** | **10** | Hyderabad | 0 | 0 | 0 |
|  | **9** | **2011** | **Sep/2011** | **South** | **11** | Coimbatore | 1 | 1 | 0 |
|  | **9** | **2011** | **Sep/2011** | **South** | **12** | Chennai | 1 | 1 | 0 |
|  | **9** | **2011** | **Sep/2011** | **South** | **13** | Trivandrum | 9 | 7 | 2 |
|  | **10** | **2011** | **Oct/2011** | **South** | **9** | Vishakhapatanam | 0 | 0 | 0 |
|  | **10** | **2011** | **Oct/2011** | **South** | **10** | Hyderabad | 0 | 0 | 0 |
|  | **10** | **2011** | **Oct/2011** | **South** | **11** | Coimbatore | 0 | 0 | 0 |
|  | **10** | **2011** | **Oct/2011** | **South** | **12** | Chennai | 3 | 2 | 1 |
|  | **10** | **2011** | **Oct/2011** | **South** | **13** | Trivandrum | 9 | 5 | 4 |
|  | **11** | **2011** | **Nov/2011** | **South** | **9** | Vishakhapatanam | 0 | 0 | 0 |
|  | **11** | **2011** | **Nov/2011** | **South** | **10** | Hyderabad | 0 | 0 | 0 |
|  | **11** | **2011** | **Nov/2011** | **South** | **11** | Coimbatore | 3 | 1 | 2 |
|  | **11** | **2011** | **Nov/2011** | **South** | **12** | Chennai | 2 | 2 | 0 |
|  | **11** | **2011** | **Nov/2011** | **South** | **13** | Trivandrum | 8 | 4 | 4 |
|  | **12** | **2011** | **Dec/2011** | **South** | **9** | Vishakhapatanam | 2 | 0 | 2 |
|  | **12** | **2011** | **Dec/2011** | **South** | **10** | Hyderabad | 0 | 0 | 0 |
|  | **12** | **2011** | **Dec/2011** | **South** | **11** | Coimbatore | 1 | 1 | 0 |
|  | **12** | **2011** | **Dec/2011** | **South** | **12** | Chennai | 0 | 0 | 0 |
|  | **12** | **2011** | **Dec/2011** | **South** | **13** | Trivandrum | 10 | 8 | 2 |
|  | **1** | **2012** | **Jan/2012** | **South** | **9** | Vishakhapatanam | 2 | 0 | 2 |
|  | **1** | **2012** | **Jan/2012** | **South** | **10** | Hyderabad | 0 | 0 | 0 |
|  | **1** | **2012** | **Jan/2012** | **South** | **11** | Coimbatore | 2 | 1 | 1 |
|  | **1** | **2012** | **Jan/2012** | **South** | **12** | Chennai | 1 | 1 | 0 |
|  | **1** | **2012** | **Jan/2012** | **South** | **13** | Trivandrum | 9 | 8 | 1 |
|  | **2** | **2012** | **Feb/2012** | **South** | **9** | Vishakhapatanam | 0 | 0 | 0 |
|  | **2** | **2012** | **Feb/2012** | **South** | **10** | Hyderabad | 0 | 0 | 0 |
|  | **2** | **2012** | **Feb/2012** | **South** | **11** | Coimbatore | 1 | 0 | 1 |
|  | **2** | **2012** | **Feb/2012** | **South** | **12** | Chennai | 5 | 3 | 2 |
|  | **2** | **2012** | **Feb/2012** | **South** | **13** | Trivandrum | 9 | 6 | 3 |
|  | **3** | **2012** | **Mar/2012** | **South** | **9** | Vishakhapatanam | 1 | 0 | 1 |
|  | **3** | **2012** | **Mar/2012** | **South** | **10** | Hyderabad | 0 | 0 | 0 |
|  | **3** | **2012** | **Mar/2012** | **South** | **11** | Coimbatore | 3 | 2 | 1 |
|  | **3** | **2012** | **Mar/2012** | **South** | **12** | Chennai | 1 | 1 | 0 |
|  | **3** | **2012** | **Mar/2012** | **South** | **13** | Trivandrum | 10 | 6 | 4 |
|  | **4** | **2012** | **Apr/2012** | **South** | **9** | Vishakhapatanam | 0 | 0 | 0 |
|  | **4** | **2012** | **Apr/2012** | **South** | **10** | Hyderabad | 0 | 0 | 0 |
|  | **4** | **2012** | **Apr/2012** | **South** | **11** | Coimbatore | 1 | 0 | 1 |
|  | **4** | **2012** | **Apr/2012** | **South** | **12** | Chennai | 5 | 5 | 0 |
|  | **4** | **2012** | **Apr/2012** | **South** | **13** | Trivandrum | 8 | 5 | 3 |
|  | **5** | **2012** | **########** | **South** | **9** | Vishakhapatanam | 0 | 0 | 0 |
|  | **5** | **2012** | **########** | **South** | **10** | Hyderabad | 0 | 0 | 0 |
|  | **5** | **2012** | **########** | **South** | **11** | Coimbatore | 2 | 1 | 1 |
|  | **5** | **2012** | **########** | **South** | **12** | Chennai | 0 | 0 | 0 |
|  | **5** | **2012** | **########** | **South** | **13** | Trivandrum | 12 | 8 | 4 |
|  | **6** | **2012** | **Jun/2012** | **South** | **9** | Vishakhapatanam | 0 | 0 | 0 |
|  | **6** | **2012** | **Jun/2012** | **South** | **10** | Hyderabad | 0 | 0 | 0 |
|  | **6** | **2012** | **Jun/2012** | **South** | **11** | Coimbatore | 0 | 0 | 0 |
|  | **6** | **2012** | **Jun/2012** | **South** | **12** | Chennai | 0 | 0 | 0 |
|  | **6** | **2012** | **Jun/2012** | **South** | **13** | Trivandrum | 7 | 3 | 4 |
|  | **7** | **2012** | **Jul/2012** | **South** | **9** | Vishakhapatanam | 2 | 1 | 1 |
|  | **7** | **2012** | **Jul/2012** | **South** | **10** | Hyderabad | 0 | 0 | 0 |
|  | **7** | **2012** | **Jul/2012** | **South** | **11** | Coimbatore | 0 | 0 | 0 |
|  | **7** | **2012** | **Jul/2012** | **South** | **12** | Chennai | 2 | 1 | 1 |
|  | **7** | **2012** | **Jul/2012** | **South** | **13** | Trivandrum | 8 | 5 | 3 |
|  | **8** | **2012** | **Aug/2012** | **South** | **9** | Vishakhapatanam | 2 | 2 | 0 |
|  | **8** | **2012** | **Aug/2012** | **South** | **10** | Hyderabad | 0 | 0 | 0 |
|  | **8** | **2012** | **Aug/2012** | **South** | **11** | Coimbatore | 3 | 2 | 1 |
|  | **8** | **2012** | **Aug/2012** | **South** | **12** | Chennai | 1 | 0 | 1 |
|  | **8** | **2012** | **Aug/2012** | **South** | **13** | Trivandrum | 4 | 2 | 2 |
|  | **9** | **2012** | **Sep/2012** | **South** | **9** | Vishakhapatanam | 2 | 1 | 1 |
|  | **9** | **2012** | **Sep/2012** | **South** | **10** | Hyderabad | 0 | 0 | 0 |
|  | **9** | **2012** | **Sep/2012** | **South** | **11** | Coimbatore | 2 | 2 | 0 |
|  | **9** | **2012** | **Sep/2012** | **South** | **12** | Chennai | 0 | 0 | 0 |
|  | **9** | **2012** | **Sep/2012** | **South** | **13** | Trivandrum | 8 | 6 | 2 |
|  | **10** | **2012** | **Oct/2012** | **South** | **9** | Vishakhapatanam | 0 | 0 | 0 |
|  | **10** | **2012** | **Oct/2012** | **South** | **10** | Hyderabad | 0 | 0 | 0 |
|  | **10** | **2012** | **Oct/2012** | **South** | **11** | Coimbatore | 2 | 0 | 2 |
|  | **10** | **2012** | **Oct/2012** | **South** | **12** | Chennai | 0 | 0 | 0 |
|  | **10** | **2012** | **Oct/2012** | **South** | **13** | Trivandrum | 6 | 2 | 4 |
|  | **11** | **2012** | **Nov/2012** | **South** | **9** | Vishakhapatanam | 0 | 0 | 0 |
|  | **11** | **2012** | **Nov/2012** | **South** | **10** | Hyderabad | 0 | 0 | 0 |
|  | **11** | **2012** | **Nov/2012** | **South** | **11** | Coimbatore | 0 | 0 | 0 |
|  | **11** | **2012** | **Nov/2012** | **South** | **12** | Chennai | 2 | 0 | 2 |
|  | **11** | **2012** | **Nov/2012** | **South** | **13** | Trivandrum | 8 | 4 | 4 |
|  | **12** | **2012** | **Dec/2012** | **South** | **9** | Vishakhapatanam | 0 | 0 | 0 |
|  | **12** | **2012** | **Dec/2012** | **South** | **10** | Hyderabad | 0 | 0 | 0 |
|  | **12** | **2012** | **Dec/2012** | **South** | **11** | Coimbatore | 0 | 0 | 0 |
|  | **12** | **2012** | **Dec/2012** | **South** | **12** | Chennai | 1 | 1 | 0 |
|  | **12** | **2012** | **Dec/2012** | **South** | **13** | Trivandrum | 5 | 3 | 2 |
|  | **1** | **2013** | **Jan/2013** | **South** | **9** | Vishakhapatanam | 0 | 0 | 0 |
|  | **1** | **2013** | **Jan/2013** | **South** | **10** | Hyderabad | 0 | 0 | 0 |
|  | **1** | **2013** | **Jan/2013** | **South** | **11** | Coimbatore | 0 | 0 | 0 |
|  | **1** | **2013** | **Jan/2013** | **South** | **12** | Chennai | 1 | 0 | 1 |
|  | **1** | **2013** | **Jan/2013** | **South** | **13** | Trivandrum | 12 | 9 | 3 |
|  | **2** | **2013** | **Feb/2013** | **South** | **9** | Vishakhapatanam | 2 | 1 | 1 |
|  | **2** | **2013** | **Feb/2013** | **South** | **10** | Hyderabad | 0 | 0 | 0 |
|  | **2** | **2013** | **Feb/2013** | **South** | **11** | Coimbatore | 0 | 0 | 0 |
|  | **2** | **2013** | **Feb/2013** | **South** | **12** | Chennai | 2 | 1 | 1 |
|  | **2** | **2013** | **Feb/2013** | **South** | **13** | Trivandrum | 12 | 8 | 4 |
|  | **3** | **2013** | **Mar/2013** | **South** | **9** | Vishakhapatanam | 1 | 0 | 1 |
|  | **3** | **2013** | **Mar/2013** | **South** | **10** | Hyderabad | 0 | 0 | 0 |
|  | **3** | **2013** | **Mar/2013** | **South** | **11** | Coimbatore | 4 | 4 | 0 |
|  | **3** | **2013** | **Mar/2013** | **South** | **12** | Chennai | 0 | 0 | 0 |
|  | **3** | **2013** | **Mar/2013** | **South** | **13** | Trivandrum | 17 | 10 | 7 |
|  | **4** | **2013** | **Apr/2013** | **South** | **9** | Vishakhapatanam | 0 | 0 | 0 |
|  | **4** | **2013** | **Apr/2013** | **South** | **10** | Hyderabad | 0 | 0 | 0 |
|  | **4** | **2013** | **Apr/2013** | **South** | **11** | Coimbatore | 1 | 1 | 0 |
|  | **4** | **2013** | **Apr/2013** | **South** | **12** | Chennai | 0 | 0 | 0 |
|  | **4** | **2013** | **Apr/2013** | **South** | **13** | Trivandrum | 14 | 9 | 5 |
|  | **5** | **2013** | **########** | **South** | **9** | Vishakhapatanam | 3 | 3 | 0 |
|  | **5** | **2013** | **########** | **South** | **10** | Hyderabad | 0 | 0 | 0 |
|  | **5** | **2013** | **########** | **South** | **11** | Coimbatore | 0 | 0 | 0 |
|  | **5** | **2013** | **########** | **South** | **12** | Chennai | 1 | 0 | 1 |
|  | **5** | **2013** | **########** | **South** | **13** | Trivandrum | 10 | 8 | 2 |
|  | **6** | **2013** | **Jun/2013** | **South** | **9** | Vishakhapatanam | 1 | 1 | 0 |
|  | **6** | **2013** | **Jun/2013** | **South** | **10** | Hyderabad | 0 | 0 | 0 |
|  | **6** | **2013** | **Jun/2013** | **South** | **11** | Coimbatore | 0 | 0 | 0 |
|  | **6** | **2013** | **Jun/2013** | **South** | **12** | Chennai | 2 | 2 | 0 |
|  | **6** | **2013** | **Jun/2013** | **South** | **13** | Trivandrum | 8 | 5 | 3 |
|  | **7** | **2013** | **Jul/2013** | **South** | **9** | Vishakhapatanam | 0 | 0 | 0 |
|  | **7** | **2013** | **Jul/2013** | **South** | **10** | Hyderabad | 0 | 0 | 0 |
|  | **7** | **2013** | **Jul/2013** | **South** | **11** | Coimbatore | 1 | 1 | 0 |
|  | **7** | **2013** | **Jul/2013** | **South** | **12** | Chennai | 0 | 0 | 0 |
|  | **7** | **2013** | **Jul/2013** | **South** | **13** | Trivandrum | 11 | 5 | 6 |
|  | **8** | **2013** | **Aug/2013** | **South** | **9** | Vishakhapatanam | 0 | 0 | 0 |
|  | **8** | **2013** | **Aug/2013** | **South** | **10** | Hyderabad | 0 | 0 | 0 |
|  | **8** | **2013** | **Aug/2013** | **South** | **11** | Coimbatore | 0 | 0 | 0 |
|  | **8** | **2013** | **Aug/2013** | **South** | **12** | Chennai | 2 | 2 | 0 |
|  | **8** | **2013** | **Aug/2013** | **South** | **13** | Trivandrum | 8 | 4 | 4 |
|  | **9** | **2013** | **Sep/2013** | **South** | **9** | Vishakhapatanam | 1 | 1 | 0 |
|  | **9** | **2013** | **Sep/2013** | **South** | **10** | Hyderabad | 0 | 0 | 0 |
|  | **9** | **2013** | **Sep/2013** | **South** | **11** | Coimbatore | 0 | 0 | 0 |
|  | **9** | **2013** | **Sep/2013** | **South** | **12** | Chennai | 2 | 2 | 0 |
|  | **9** | **2013** | **Sep/2013** | **South** | **13** | Trivandrum | 6 | 4 | 2 |
|  | **10** | **2013** | **Oct/2013** | **South** | **9** | Vishakhapatanam | 3 | 3 | 0 |
|  | **10** | **2013** | **Oct/2013** | **South** | **10** | Hyderabad | 0 | 0 | 0 |
|  | **10** | **2013** | **Oct/2013** | **South** | **11** | Coimbatore | 4 | 1 | 3 |
|  | **10** | **2013** | **Oct/2013** | **South** | **12** | Chennai | 0 | 0 | 0 |
|  | **10** | **2013** | **Oct/2013** | **South** | **13** | Trivandrum | 3 | 3 | 0 |
|  | **11** | **2013** | **Nov/2013** | **South** | **9** | Vishakhapatanam | 0 | 0 | 0 |
|  | **11** | **2013** | **Nov/2013** | **South** | **10** | Hyderabad | 0 | 0 | 0 |
|  | **11** | **2013** | **Nov/2013** | **South** | **11** | Coimbatore | 0 | 0 | 0 |
|  | **11** | **2013** | **Nov/2013** | **South** | **12** | Chennai | 4 | 1 | 3 |
|  | **11** | **2013** | **Nov/2013** | **South** | **13** | Trivandrum | 8 | 7 | 1 |
|  | **12** | **2013** | **Dec/2013** | **South** | **9** | Vishakhapatanam | 1 | 0 | 1 |
|  | **12** | **2013** | **Dec/2013** | **South** | **10** | Hyderabad | 0 | 0 | 0 |
|  | **12** | **2013** | **Dec/2013** | **South** | **11** | Coimbatore | 3 | 1 | 2 |
|  | **12** | **2013** | **Dec/2013** | **South** | **12** | Chennai | 3 | 2 | 1 |
|  | **12** | **2013** | **Dec/2013** | **South** | **13** | Trivandrum | 13 | 5 | 8 |
|  | **1** | **2014** | **Jan/2014** | **South** | **9** | Vishakhapatanam | 2 | 1 | 1 |
|  | **1** | **2014** | **Jan/2014** | **South** | **10** | Hyderabad | 0 | 0 | 0 |
|  | **1** | **2014** | **Jan/2014** | **South** | **11** | Coimbatore | 1 | 1 | 0 |
|  | **1** | **2014** | **Jan/2014** | **South** | **12** | Chennai | 0 | 0 | 0 |
|  | **1** | **2014** | **Jan/2014** | **South** | **13** | Trivandrum | 10 | 5 | 5 |
|  | **2** | **2014** | **Feb/2014** | **South** | **9** | Vishakhapatanam | 0 | 0 | 0 |
|  | **2** | **2014** | **Feb/2014** | **South** | **10** | Hyderabad | 0 | 0 | 0 |
|  | **2** | **2014** | **Feb/2014** | **South** | **11** | Coimbatore | 1 | 1 | 0 |
|  | **2** | **2014** | **Feb/2014** | **South** | **12** | Chennai | 0 | 0 | 0 |
|  | **2** | **2014** | **Feb/2014** | **South** | **13** | Trivandrum | 7 | 3 | 4 |
|  | **3** | **2014** | **Mar/2014** | **South** | **9** | Vishakhapatanam | 1 | 1 | 0 |
|  | **3** | **2014** | **Mar/2014** | **South** | **10** | Hyderabad | 1 | 0 | 1 |
|  | **3** | **2014** | **Mar/2014** | **South** | **11** | Coimbatore | 2 | 2 | 0 |
|  | **3** | **2014** | **Mar/2014** | **South** | **12** | Chennai | 0 | 0 | 0 |
|  | **3** | **2014** | **Mar/2014** | **South** | **13** | Trivandrum | 10 | 5 | 5 |
|  | **4** | **2014** | **Apr/2014** | **South** | **9** | Vishakhapatanam | 1 | 0 | 1 |
|  | **4** | **2014** | **Apr/2014** | **South** | **10** | Hyderabad | 0 | 0 | 0 |
|  | **4** | **2014** | **Apr/2014** | **South** | **11** | Coimbatore | 1 | 1 | 0 |
|  | **4** | **2014** | **Apr/2014** | **South** | **12** | Chennai | 2 | 0 | 2 |
|  | **4** | **2014** | **Apr/2014** | **South** | **13** | Trivandrum | 2 | 2 | 0 |
|  | **5** | **2014** | **########** | **South** | **9** | Vishakhapatanam | 0 | 0 | 0 |
|  | **5** | **2014** | **########** | **South** | **10** | Hyderabad | 0 | 0 | 0 |
|  | **5** | **2014** | **########** | **South** | **11** | Coimbatore | 2 | 1 | 1 |
|  | **5** | **2014** | **########** | **South** | **12** | Chennai | 0 | 0 | 0 |
|  | **5** | **2014** | **########** | **South** | **13** | Trivandrum | 9 | 4 | 5 |
|  | **6** | **2014** | **Jun/2014** | **South** | **9** | Vishakhapatanam | 0 | 0 | 0 |
|  | **6** | **2014** | **Jun/2014** | **South** | **10** | Hyderabad | 0 | 0 | 0 |
|  | **6** | **2014** | **Jun/2014** | **South** | **11** | Coimbatore | 0 | 0 | 0 |
|  | **6** | **2014** | **Jun/2014** | **South** | **12** | Chennai | 1 | 1 | 0 |
|  | **6** | **2014** | **Jun/2014** | **South** | **13** | Trivandrum | 12 | 11 | 1 |
|  | **7** | **2014** | **Jul/2014** | **South** | **9** | Vishakhapatanam | 0 | 0 | 0 |
|  | **7** | **2014** | **Jul/2014** | **South** | **10** | Hyderabad | 0 | 0 | 0 |
|  | **7** | **2014** | **Jul/2014** | **South** | **11** | Coimbatore | 0 | 0 | 0 |
|  | **7** | **2014** | **Jul/2014** | **South** | **12** | Chennai | 0 | 0 | 0 |
|  | **7** | **2014** | **Jul/2014** | **South** | **13** | Trivandrum | 7 | 3 | 4 |
|  | **8** | **2014** | **Aug/2014** | **South** | **9** | Vishakhapatanam | 1 | 1 | 0 |
|  | **8** | **2014** | **Aug/2014** | **South** | **10** | Hyderabad | 1 | 1 | 0 |
|  | **8** | **2014** | **Aug/2014** | **South** | **11** | Coimbatore | 0 | 0 | 0 |
|  | **8** | **2014** | **Aug/2014** | **South** | **12** | Chennai | 0 | 0 | 0 |
|  | **8** | **2014** | **Aug/2014** | **South** | **13** | Trivandrum | 4 | 3 | 1 |
|  | **9** | **2014** | **Sep/2014** | **South** | **9** | Vishakhapatanam | 0 | 0 | 0 |
|  | **9** | **2014** | **Sep/2014** | **South** | **10** | Hyderabad | 0 | 0 | 0 |
|  | **9** | **2014** | **Sep/2014** | **South** | **11** | Coimbatore | 0 | 0 | 0 |
|  | **9** | **2014** | **Sep/2014** | **South** | **12** | Chennai | 1 | 1 | 0 |
|  | **9** | **2014** | **Sep/2014** | **South** | **13** | Trivandrum | 6 | 6 | 0 |
|  | **10** | **2014** | **Oct/2014** | **South** | **9** | Vishakhapatanam | 1 | 0 | 1 |
|  | **10** | **2014** | **Oct/2014** | **South** | **10** | Hyderabad | 0 | 0 | 0 |
|  | **10** | **2014** | **Oct/2014** | **South** | **11** | Coimbatore | 3 | 2 | 1 |
|  | **10** | **2014** | **Oct/2014** | **South** | **12** | Chennai | 2 | 2 | 0 |
|  | **10** | **2014** | **Oct/2014** | **South** | **13** | Trivandrum | 16 | 11 | 5 |
|  | **11** | **2014** | **Nov/2014** | **South** | **9** | Vishakhapatanam | 1 | 1 | 0 |
|  | **11** | **2014** | **Nov/2014** | **South** | **10** | Hyderabad | 0 | 0 | 0 |
|  | **11** | **2014** | **Nov/2014** | **South** | **11** | Coimbatore | 3 | 1 | 2 |
|  | **11** | **2014** | **Nov/2014** | **South** | **12** | Chennai | 2 | 1 | 1 |
|  | **11** | **2014** | **Nov/2014** | **South** | **13** | Trivandrum | 6 | 3 | 3 |
|  | **12** | **2014** | **Dec/2014** | **South** | **9** | Vishakhapatanam | 0 | 0 | 0 |
|  | **12** | **2014** | **Dec/2014** | **South** | **10** | Hyderabad | 0 | 0 | 0 |
|  | **12** | **2014** | **Dec/2014** | **South** | **11** | Coimbatore | 2 | 2 | 0 |
|  | **12** | **2014** | **Dec/2014** | **South** | **12** | Chennai | 1 | 0 | 1 |
|  | **12** | **2014** | **Dec/2014** | **South** | **13** | Trivandrum | 14 | 10 | 4 |
|  | **1** | **2015** | **Jan/2015** | **South** | **9** | Vishakhapatanam | 1 | 0 | 1 |
|  | **1** | **2015** | **Jan/2015** | **South** | **10** | Hyderabad | 0 | 0 | 0 |
|  | **1** | **2015** | **Jan/2015** | **South** | **11** | Coimbatore | 2 | 0 | 2 |
|  | **1** | **2015** | **Jan/2015** | **South** | **12** | Chennai | 0 | 0 | 0 |
|  | **1** | **2015** | **Jan/2015** | **South** | **13** | Trivandrum | 16 | 11 | 5 |
|  | **2** | **2015** | **Feb/2015** | **South** | **9** | Vishakhapatanam | 0 | 0 | 0 |
|  | **2** | **2015** | **Feb/2015** | **South** | **10** | Hyderabad | 0 | 0 | 0 |
|  | **2** | **2015** | **Feb/2015** | **South** | **11** | Coimbatore | 3 | 1 | 2 |
|  | **2** | **2015** | **Feb/2015** | **South** | **12** | Chennai | 0 | 0 | 0 |
|  | **2** | **2015** | **Feb/2015** | **South** | **13** | Trivandrum | 6 | 5 | 1 |
|  | **3** | **2015** | **Mar/2015** | **South** | **9** | Vishakhapatanam | 2 | 0 | 2 |
|  | **3** | **2015** | **Mar/2015** | **South** | **10** | Hyderabad | 0 | 0 | 0 |
|  | **3** | **2015** | **Mar/2015** | **South** | **11** | Coimbatore | 2 | 2 | 0 |
|  | **3** | **2015** | **Mar/2015** | **South** | **12** | Chennai | 1 | 1 | 0 |
|  | **3** | **2015** | **Mar/2015** | **South** | **13** | Trivandrum | 12 | 8 | 4 |
|  | **4** | **2015** | **Apr/2015** | **South** | **9** | Vishakhapatanam | 0 | 0 | 0 |
|  | **4** | **2015** | **Apr/2015** | **South** | **10** | Hyderabad | 0 | 0 | 0 |
|  | **4** | **2015** | **Apr/2015** | **South** | **11** | Coimbatore | 3 | 2 | 1 |
|  | **4** | **2015** | **Apr/2015** | **South** | **12** | Chennai | 0 | 0 | 0 |
|  | **4** | **2015** | **Apr/2015** | **South** | **13** | Trivandrum | 8 | 6 | 2 |
|  | **5** | **2015** | **########** | **South** | **9** | Vishakhapatanam | 1 | 0 | 1 |
|  | **5** | **2015** | **########** | **South** | **10** | Hyderabad | 0 | 0 | 0 |
|  | **5** | **2015** | **########** | **South** | **11** | Coimbatore | 1 | 0 | 1 |
|  | **5** | **2015** | **########** | **South** | **12** | Chennai | 3 | 2 | 1 |
|  | **5** | **2015** | **########** | **South** | **13** | Trivandrum | 14 | 9 | 5 |
|  | **6** | **2015** | **Jun/2015** | **South** | **9** | Vishakhapatanam | 0 | 0 | 0 |
|  | **6** | **2015** | **Jun/2015** | **South** | **10** | Hyderabad | 0 | 0 | 0 |
|  | **6** | **2015** | **Jun/2015** | **South** | **11** | Coimbatore | 1 | 1 | 0 |
|  | **6** | **2015** | **Jun/2015** | **South** | **12** | Chennai | 1 | 0 | 1 |
|  | **6** | **2015** | **Jun/2015** | **South** | **13** | Trivandrum | 20 | 13 | 7 |
|  | **7** | **2015** | **Jul/2015** | **South** | **9** | Vishakhapatanam | 1 | 0 | 1 |
|  | **7** | **2015** | **Jul/2015** | **South** | **10** | Hyderabad | 0 | 0 | 0 |
|  | **7** | **2015** | **Jul/2015** | **South** | **11** | Coimbatore | 0 | 0 | 0 |
|  | **7** | **2015** | **Jul/2015** | **South** | **12** | Chennai | 1 | 1 | 0 |
|  | **7** | **2015** | **Jul/2015** | **South** | **13** | Trivandrum | 22 | 10 | 12 |
|  | **8** | **2015** | **Aug/2015** | **South** | **9** | Vishakhapatanam | 0 | 0 | 0 |
|  | **8** | **2015** | **Aug/2015** | **South** | **10** | Hyderabad | 0 | 0 | 0 |
|  | **8** | **2015** | **Aug/2015** | **South** | **11** | Coimbatore | 1 | 1 | 0 |
|  | **8** | **2015** | **Aug/2015** | **South** | **12** | Chennai | 0 | 0 | 0 |
|  | **8** | **2015** | **Aug/2015** | **South** | **13** | Trivandrum | 11 | 6 | 5 |
|  | **9** | **2015** | **Sep/2015** | **South** | **9** | Vishakhapatanam | 3 | 2 | 1 |
|  | **9** | **2015** | **Sep/2015** | **South** | **10** | Hyderabad | 0 | 0 | 0 |
|  | **9** | **2015** | **Sep/2015** | **South** | **11** | Coimbatore | 0 | 0 | 0 |
|  | **9** | **2015** | **Sep/2015** | **South** | **12** | Chennai | 0 | 0 | 0 |
|  | **9** | **2015** | **Sep/2015** | **South** | **13** | Trivandrum | 8 | 6 | 2 |
|  | **10** | **2015** | **Oct/2015** | **South** | **9** | Vishakhapatanam | 1 | 1 | 0 |
|  | **10** | **2015** | **Oct/2015** | **South** | **10** | Hyderabad | 0 | 0 | 0 |
|  | **10** | **2015** | **Oct/2015** | **South** | **11** | Coimbatore | 0 | 0 | 0 |
|  | **10** | **2015** | **Oct/2015** | **South** | **12** | Chennai | 0 | 0 | 0 |
|  | **10** | **2015** | **Oct/2015** | **South** | **13** | Trivandrum | 12 | 8 | 4 |
|  | **11** | **2015** | **Nov/2015** | **South** | **9** | Vishakhapatanam | 1 | 1 | 0 |
|  | **11** | **2015** | **Nov/2015** | **South** | **10** | Hyderabad | 1 | 1 | 0 |
|  | **11** | **2015** | **Nov/2015** | **South** | **11** | Coimbatore | 0 | 0 | 0 |
|  | **11** | **2015** | **Nov/2015** | **South** | **12** | Chennai | 1 | 0 | 1 |
|  | **11** | **2015** | **Nov/2015** | **South** | **13** | Trivandrum | 9 | 6 | 3 |
|  | **12** | **2015** | **Dec/2015** | **South** | **9** | Vishakhapatanam | 2 | 1 | 1 |
|  | **12** | **2015** | **Dec/2015** | **South** | **10** | Hyderabad | 0 | 0 | 0 |
|  | **12** | **2015** | **Dec/2015** | **South** | **11** | Coimbatore | 2 | 1 | 1 |
|  | **12** | **2015** | **Dec/2015** | **South** | **12** | Chennai | 0 | 0 | 0 |
|  | **12** | **2015** | **Dec/2015** | **South** | **13** | Trivandrum | 12 | 9 | 3 |
|  | **1** | **2016** | **Jan/2016** | **South** | **9** | Vishakhapatanam | 0 | 0 | 0 |
|  | **1** | **2016** | **Jan/2016** | **South** | **10** | Hyderabad | 0 | 0 | 0 |
|  | **1** | **2016** | **Jan/2016** | **South** | **11** | Coimbatore | 1 | 1 | 0 |
|  | **1** | **2016** | **Jan/2016** | **South** | **12** | Chennai | 0 | 0 | 0 |
|  | **1** | **2016** | **Jan/2016** | **South** | **13** | Trivandrum | 8 | 5 | 3 |
|  | **2** | **2016** | **Feb/2016** | **South** | **9** | Vishakhapatanam | 1 | 1 | 0 |
|  | **2** | **2016** | **Feb/2016** | **South** | **10** | Hyderabad | 0 | 0 | 0 |
|  | **2** | **2016** | **Feb/2016** | **South** | **11** | Coimbatore | 1 | 0 | 1 |
|  | **2** | **2016** | **Feb/2016** | **South** | **12** | Chennai | 0 | 0 | 0 |
|  | **2** | **2016** | **Feb/2016** | **South** | **13** | Trivandrum | 10 | 7 | 3 |
|  | **3** | **2016** | **Mar/2016** | **South** | **9** | Vishakhapatanam | 3 | 0 | 3 |
|  | **3** | **2016** | **Mar/2016** | **South** | **10** | Hyderabad | 0 | 0 | 0 |
|  | **3** | **2016** | **Mar/2016** | **South** | **11** | Coimbatore | 1 | 0 | 1 |
|  | **3** | **2016** | **Mar/2016** | **South** | **12** | Chennai | 0 | 0 | 0 |
|  | **3** | **2016** | **Mar/2016** | **South** | **13** | Trivandrum | 15 | 11 | 4 |
|  | **4** | **2016** | **Apr/2016** | **South** | **9** | Vishakhapatanam | 2 | 1 | 1 |
|  | **4** | **2016** | **Apr/2016** | **South** | **10** | Hyderabad | 0 | 0 | 0 |
|  | **4** | **2016** | **Apr/2016** | **South** | **11** | Coimbatore | 1 | 1 | 0 |
|  | **4** | **2016** | **Apr/2016** | **South** | **12** | Chennai | 1 | 1 | 0 |
|  | **4** | **2016** | **Apr/2016** | **South** | **13** | Trivandrum | 17 | 13 | 4 |
|  | **5** | **2016** | **########** | **South** | **9** | Vishakhapatanam | 2 | 2 | 0 |
|  | **5** | **2016** | **########** | **South** | **10** | Hyderabad | 0 | 0 | 0 |
|  | **5** | **2016** | **########** | **South** | **11** | Coimbatore | 2 | 1 | 1 |
|  | **5** | **2016** | **########** | **South** | **12** | Chennai | 1 | 0 | 1 |
|  | **5** | **2016** | **########** | **South** | **13** | Trivandrum | 11 | 7 | 4 |
|  | **6** | **2016** | **Jun/2016** | **South** | **9** | Vishakhapatanam | 2 | 1 | 1 |
|  | **6** | **2016** | **Jun/2016** | **South** | **10** | Hyderabad | 0 | 0 | 0 |
|  | **6** | **2016** | **Jun/2016** | **South** | **11** | Coimbatore | 1 | 0 | 1 |
|  | **6** | **2016** | **Jun/2016** | **South** | **12** | Chennai | 0 | 0 | 0 |
|  | **6** | **2016** | **Jun/2016** | **South** | **13** | Trivandrum | 10 | 8 | 2 |
|  | **7** | **2016** | **Jul/2016** | **South** | **9** | Vishakhapatanam | 3 | 2 | 1 |
|  | **7** | **2016** | **Jul/2016** | **South** | **10** | Hyderabad | 0 | 0 | 0 |
|  | **7** | **2016** | **Jul/2016** | **South** | **11** | Coimbatore | 3 | 2 | 1 |
|  | **7** | **2016** | **Jul/2016** | **South** | **12** | Chennai | 4 | 2 | 2 |
|  | **7** | **2016** | **Jul/2016** | **South** | **13** | Trivandrum | 11 | 7 | 4 |
|  | **8** | **2016** | **Aug/2016** | **South** | **9** | Vishakhapatanam | 1 | 1 | 0 |
|  | **8** | **2016** | **Aug/2016** | **South** | **10** | Hyderabad | 0 | 0 | 0 |
|  | **8** | **2016** | **Aug/2016** | **South** | **11** | Coimbatore | 2 | 1 | 1 |
|  | **8** | **2016** | **Aug/2016** | **South** | **12** | Chennai | 1 | 1 | 0 |
|  | **8** | **2016** | **Aug/2016** | **South** | **13** | Trivandrum | 9 | 5 | 4 |
|  | **9** | **2016** | **Sep/2016** | **South** | **9** | Vishakhapatanam | 1 | 0 | 1 |
|  | **9** | **2016** | **Sep/2016** | **South** | **10** | Hyderabad | 0 | 0 | 0 |
|  | **9** | **2016** | **Sep/2016** | **South** | **11** | Coimbatore | 4 | 3 | 1 |
|  | **9** | **2016** | **Sep/2016** | **South** | **12** | Chennai | 2 | 1 | 1 |
|  | **9** | **2016** | **Sep/2016** | **South** | **13** | Trivandrum | 9 | 5 | 4 |
|  | **10** | **2016** | **Oct/2016** | **South** | **9** | Vishakhapatanam | 2 | 2 | 0 |
|  | **10** | **2016** | **Oct/2016** | **South** | **10** | Hyderabad | 0 | 0 | 0 |
|  | **10** | **2016** | **Oct/2016** | **South** | **11** | Coimbatore | 1 | 1 | 0 |
|  | **10** | **2016** | **Oct/2016** | **South** | **12** | Chennai | 1 | 1 | 0 |
|  | **10** | **2016** | **Oct/2016** | **South** | **13** | Trivandrum | 5 | 3 | 2 |
|  | **11** | **2016** | **Nov/2016** | **South** | **9** | Vishakhapatanam | 4 | 1 | 3 |
|  | **11** | **2016** | **Nov/2016** | **South** | **10** | Hyderabad | 0 | 0 | 0 |
|  | **11** | **2016** | **Nov/2016** | **South** | **11** | Coimbatore | 0 | 0 | 0 |
|  | **11** | **2016** | **Nov/2016** | **South** | **12** | Chennai | 0 | 0 | 0 |
|  | **11** | **2016** | **Nov/2016** | **South** | **13** | Trivandrum | 8 | 7 | 1 |
|  | **12** | **2016** | **Dec/2016** | **South** | **9** | Vishakhapatanam | 0 | 0 | 0 |
|  | **12** | **2016** | **Dec/2016** | **South** | **10** | Hyderabad | 0 | 0 | 0 |
|  | **12** | **2016** | **Dec/2016** | **South** | **11** | Coimbatore | 1 | 1 | 0 |
|  | **12** | **2016** | **Dec/2016** | **South** | **12** | Chennai | 0 | 0 | 0 |
|  | **12** | **2016** | **Dec/2016** | **South** | **13** | Trivandrum | 12 | 5 | 7 |
|  | **1** | **2017** | **Jan/2017** | **South** | **9** | Vishakhapatanam | 1 | 1 | 0 |
|  | **1** | **2017** | **Jan/2017** | **South** | **10** | Hyderabad | 0 | 0 | 0 |
|  | **1** | **2017** | **Jan/2017** | **South** | **11** | Coimbatore | 3 | 2 | 1 |
|  | **1** | **2017** | **Jan/2017** | **South** | **12** | Chennai | 0 | 0 | 0 |
|  | **1** | **2017** | **Jan/2017** | **South** | **13** | Trivandrum | 9 | 5 | 4 |
|  | **2** | **2017** | **Feb/2017** | **South** | **9** | Vishakhapatanam | 0 | 0 | 0 |
|  | **2** | **2017** | **Feb/2017** | **South** | **10** | Hyderabad | 0 | 0 | 0 |
|  | **2** | **2017** | **Feb/2017** | **South** | **11** | Coimbatore | 2 | 2 | 0 |
|  | **2** | **2017** | **Feb/2017** | **South** | **12** | Chennai | 0 | 0 | 0 |
|  | **2** | **2017** | **Feb/2017** | **South** | **13** | Trivandrum | 13 | 9 | 4 |
|  | **3** | **2017** | **Mar/2017** | **South** | **9** | Vishakhapatanam | 1 | 1 | 0 |
|  | **3** | **2017** | **Mar/2017** | **South** | **10** | Hyderabad | 0 | 0 | 0 |
|  | **3** | **2017** | **Mar/2017** | **South** | **11** | Coimbatore | 3 | 2 | 1 |
|  | **3** | **2017** | **Mar/2017** | **South** | **12** | Chennai | 0 | 0 | 0 |
|  | **3** | **2017** | **Mar/2017** | **South** | **13** | Trivandrum | 18 | 12 | 6 |
|  | **4** | **2017** | **Apr/2017** | **South** | **9** | Vishakhapatanam | 1 | 0 | 1 |
|  | **4** | **2017** | **Apr/2017** | **South** | **10** | Hyderabad | 0 | 0 | 0 |
|  | **4** | **2017** | **Apr/2017** | **South** | **11** | Coimbatore | 0 | 0 | 0 |
|  | **4** | **2017** | **Apr/2017** | **South** | **12** | Chennai | 1 | 0 | 1 |
|  | **4** | **2017** | **Apr/2017** | **South** | **13** | Trivandrum | 11 | 6 | 5 |
|  | **5** | **2017** | **########** | **South** | **9** | Vishakhapatanam | 1 | 1 | 0 |
|  | **5** | **2017** | **########** | **South** | **10** | Hyderabad | 0 | 0 | 0 |
|  | **5** | **2017** | **########** | **South** | **11** | Coimbatore | 2 | 0 | 2 |
|  | **5** | **2017** | **########** | **South** | **12** | Chennai | 3 | 2 | 1 |
|  | **5** | **2017** | **########** | **South** | **13** | Trivandrum | 15 | 13 | 2 |
|  | **6** | **2017** | **Jun/2017** | **South** | **9** | Vishakhapatanam | 2 | 2 | 0 |
|  | **6** | **2017** | **Jun/2017** | **South** | **10** | Hyderabad | 0 | 0 | 0 |
|  | **6** | **2017** | **Jun/2017** | **South** | **11** | Coimbatore | 2 | 2 | 0 |
|  | **6** | **2017** | **Jun/2017** | **South** | **12** | Chennai | 2 | 0 | 2 |
|  | **6** | **2017** | **Jun/2017** | **South** | **13** | Trivandrum | 10 | 5 | 5 |
|  | **7** | **2017** | **Jul/2017** | **South** | **9** | Vishakhapatanam | 1 | 1 | 0 |
|  | **7** | **2017** | **Jul/2017** | **South** | **10** | Hyderabad | 0 | 0 | 0 |
|  | **7** | **2017** | **Jul/2017** | **South** | **11** | Coimbatore | 0 | 0 | 0 |
|  | **7** | **2017** | **Jul/2017** | **South** | **12** | Chennai | 1 | 1 | 0 |
|  | **7** | **2017** | **Jul/2017** | **South** | **13** | Trivandrum | 10 | 8 | 2 |
|  | **8** | **2017** | **Aug/2017** | **South** | **9** | Vishakhapatanam | 0 | 0 | 0 |
|  | **8** | **2017** | **Aug/2017** | **South** | **10** | Hyderabad | 0 | 0 | 0 |
|  | **8** | **2017** | **Aug/2017** | **South** | **11** | Coimbatore | 1 | 0 | 1 |
|  | **8** | **2017** | **Aug/2017** | **South** | **12** | Chennai | 1 | 0 | 1 |
|  | **8** | **2017** | **Aug/2017** | **South** | **13** | Trivandrum | 7 | 5 | 2 |
|  | **9** | **2017** | **Sep/2017** | **South** | **9** | Vishakhapatanam | 1 | 0 | 1 |
|  | **9** | **2017** | **Sep/2017** | **South** | **10** | Hyderabad | 0 | 0 | 0 |
|  | **9** | **2017** | **Sep/2017** | **South** | **11** | Coimbatore | 0 | 0 | 0 |
|  | **9** | **2017** | **Sep/2017** | **South** | **12** | Chennai | 0 | 0 | 0 |
|  | **9** | **2017** | **Sep/2017** | **South** | **13** | Trivandrum | 6 | 4 | 2 |
|  | **5** | **2016** | **########** | **South** | **11** | Coimbatore | 3 | 3 | 0 |
|  | **6** | **2016** | **Jun/2016** | **South** | **11** | Coimbatore | 1 | 0 | 1 |
|  | **7** | **2016** | **Jul/2016** | **South** | **11** | Coimbatore | 2 | 1 | 1 |
|  | **8** | **2016** | **Aug/2016** | **South** | **11** | Coimbatore | 0 | 0 | 0 |
|  | **9** | **2016** | **Sep/2016** | **South** | **11** | Coimbatore | 0 | 0 | 0 |
|  | **10** | **2016** | **Oct/2016** | **South** | **11** | Coimbatore | 1 | 1 | 0 |
|  | **11** | **2016** | **Nov/2016** | **South** | **11** | Coimbatore | 2 | 2 | 0 |
|  | **12** | **2016** | **Dec/2016** | **South** | **11** | Coimbatore | 0 | 0 | 0 |
|  | **1** | **2017** | **Jan/2017** | **South** | **11** | Coimbatore | 1 | 0 | 1 |
|  | **3** | **2017** | **Mar/2017** | **South** | **11** | Coimbatore | 1 | 0 | 1 |
|  | **4** | **2017** | **Apr/2017** | **South** | **11** | Coimbatore | 1 | 1 | 0 |
|  | **5** | **2017** | **########** | **South** | **11** | Coimbatore | 0 | 0 | 0 |
|  | **7** | **2017** | **Jul/2017** | **South** | **11** | Coimbatore | 0 | 0 | 0 |
|  | **9** | **2017** | **Sep/2017** | **South** | **11** | Coimbatore | 0 | 0 | 0 |
|  | **5** | **2016** | **########** | **South** | **11** | Coimbatore | 1 | 0 | 1 |
|  | **6** | **2016** | **Jun/2016** | **South** | **11** | Coimbatore | 1 | 1 | 0 |
|  | **7** | **2016** | **Jul/2016** | **South** | **11** | Coimbatore | 0 | 0 | 0 |
|  | **8** | **2016** | **Aug/2016** | **South** | **11** | Coimbatore | 5 | 3 | 2 |
|  | **9** | **2016** | **Sep/2016** | **South** | **11** | Coimbatore | 2 | 0 | 2 |
|  | **10** | **2016** | **Oct/2016** | **South** | **11** | Coimbatore | 2 | 1 | 1 |
|  | **11** | **2016** | **Nov/2016** | **South** | **11** | Coimbatore | 1 | 1 | 0 |
|  | **12** | **2016** | **Dec/2016** | **South** | **11** | Coimbatore | 1 | 1 | 0 |
|  | **1** | **2017** | **Jan/2017** | **South** | **11** | Coimbatore | 1 | 0 | 1 |
|  | **3** | **2017** | **Mar/2017** | **South** | **11** | Coimbatore | 1 | 0 | 1 |
|  | **4** | **2017** | **Apr/2017** | **South** | **11** | Coimbatore | 1 | 1 | 0 |
|  | **5** | **2017** | **########** | **South** | **11** | Coimbatore | 2 | 0 | 2 |
|  | **7** | **2017** | **Jul/2017** | **South** | **11** | Coimbatore | 0 | 0 | 0 |
|  | **9** | **2017** | **Sep/2017** | **South** | **11** | Coimbatore | 1 | 0 | 1 |
|  | **5** | **2016** | **########** | **South** | **11** | Coimbatore | 0 | 0 | 0 |
|  | **6** | **2016** | **Jun/2016** | **South** | **11** | Coimbatore | 1 | 1 | 0 |
|  | **7** | **2016** | **Jul/2016** | **South** | **11** | Coimbatore | 1 | 0 | 1 |
|  | **8** | **2016** | **Aug/2016** | **South** | **11** | Coimbatore | 0 | 0 | 0 |
|  | **9** | **2016** | **Sep/2016** | **South** | **11** | Coimbatore | 0 | 0 | 0 |
|  | **10** | **2016** | **Oct/2016** | **South** | **11** | Coimbatore | 0 | 0 | 0 |
|  | **11** | **2016** | **Nov/2016** | **South** | **11** | Coimbatore | 1 | 1 | 0 |
|  | **12** | **2016** | **Dec/2016** | **South** | **11** | Coimbatore | 0 | 0 | 0 |
|  | **1** | **2017** | **Jan/2017** | **South** | **11** | Coimbatore | 1 | 1 | 0 |
|  | **3** | **2017** | **Mar/2017** | **South** | **11** | Coimbatore | 2 | 1 | 1 |
|  | **4** | **2017** | **Apr/2017** | **South** | **11** | Coimbatore | 1 | 1 | 0 |
|  | **5** | **2017** | **########** | **South** | **11** | Coimbatore | 0 | 0 | 0 |
|  | **7** | **2017** | **Jul/2017** | **South** | **11** | Coimbatore | 2 | 1 | 1 |
|  | **9** | **2017** | **Sep/2017** | **South** | **11** | Coimbatore | 0 | 0 | 0 |

**Supplementary Figure S3: Temporal distribution of hospital reported intussusception cases – 2010- 2017**
